# Mental Health Status of Women With Rheumatoid Arthritis in Iran

**DOI:** 10.5812/ircmj.14250

**Published:** 2014-02-02

**Authors:** Sousan Kolahi, Hamid Noshad, Ali Fakhari, Ali Reza Khabbazi, Mehrzad Hajaliloo, Leila Ghahremani Nasab

**Affiliations:** 1 Connective Tissue Diseases Research Center, Tabriz University of Medical Sciences, Tabriz, IR Iran; 2 Chronic Kidney Diseases Research Center, Tabriz University of Medical Sciences, Tabriz, IR Iran; 3 Department of Psychiatry, Tabriz University of Medical Sciences, Tabriz, IR Iran

**Keywords:** Arthritis, Mental Health, Iran

## Abstract

**Background:**

Chronic diseases are usually accompanied by psychological abnormalities. Anxiety and depression occur in a significant number of patients with rheumatoid arthritis (RA). These psychological problems are likely, to be the results of chronic physical symptoms such as pain and disability.

**Objectives:**

The aim of this study was the evaluation of mental health in patients with rheumatoid arthritis in Iran.

**Patients and Methods:**

One hundred women with definite diagnosis of RA were evaluated in the outpatient clinic of the Tabriz University of Medical Sciences during one year period. Activity of RA disease was determined according to the Disease Activity Score-28 (DAS-28) scaling system and mental health was evaluated using the General Health Questionnaire-28 (GHQ-28). Based on the cut of point score of 22, prevalence of psychological problems was determined and a comparison was made the between two groups (with and without psychological problems).

**Results:**

GHQ28 screening test showed that psychological problems were seen in 49% of studied patients. There were significant difference between duration of disease and DAS-28 score between the two groups (P = 0.001 and P = 0.001, respectively). Somatic symptoms were more frequent in patients with psychological problems (P = 0.001). Somatic symptoms in patient with high disease activity was also more frequent than the other group (P = 0.002). There was a significant positive correlation between the scores of DAS-28 and GHQ-28 (r = 0.329, P = 0.001).

**Conclusions:**

This study showed that a considerable portion of patients with RA may have mental problems. The probability of these problems increased with more severe and more prolonged disease.

## 1. Background

Rheumatoid arthritis (RA) is a chronic polyarticular disease. It leads to articular damage, deformity and pain. It is an autoimmune disease that results in a chronic, systemic inflammatory disorder that may affect many tissues and organs, but principally attacks flexible (synovial) joints. It can be a disabling and painful condition, which can lead to substantial loss of functioning and mobility if not adequately treated. RA, as a chronic disease has several disabling complications and psychological problems (1). Social and psychological problems are frequently seen in patients with RA. On the other hand it is associated with high mortality rate (2, 3). In a way, depression can be regarded as an inevitable component of living with chronic diseases. This will directly affect management and treatment of patients with RA. Mental health is defined as state of mind characterized by enthusiasm for life, relative absence of anxiety and other symptoms, capacity for establishing constructive relationships and overcoming daily desires and tensions (4). A concept close to mental health is subjective well-being which implies positive feelings and satisfaction with life including satisfaction with self and others in the social domains such as family, job, etc. Previous studies have examined the relationship between mental health level and subjective well-being (5).

The quality of life, mental health status, and family dysfunction studies in Colombian patients with rheumatoid arthritis showed that all of these factors are affected with disease activity (6). People who live with chronic diseases such as rheumatoid arthritis may experience more than just physical pain and disability. Their illness could also affect their mental health (7). Relation between mental health status and all-cause mortality in patients with RA was studied and it was shown that mental health and morbidity affects the disease activity and duration (8). These patients more likely stop taking their medications when compared to RA patients without depression (9). In a Korean study, it was shown that the level of daily performance and depression, rather than disease activity and pain, have more profound effect on health related quality of life (HRQoL) and mental health status in patients with RA (10). In the Moroccan patients with rheumatoid arthritis, health-related quality of life and mental health were affected by disease duration and activity (11). Early RA has shown to have a broad impact on HRQoL in South Africans, with a large proportion of patients still showing substantial functional disability and suboptimal mental health after 12 months of pharmacological therapy (12). RA is the most common chronic rheumatologic disorder and chronic pain, functional impairment and social stress along with social isolation may lead to psychological problems (13). There are many published studies of depression in RA patients (14, 15) but only few of them were designed according to the standardized interview styles.

## 2. Objectives

We could not find any Iranian study examining psychiatric disorders in RA patients using standardized psychiatric interview. The current study was designed to determine the psychiatric problems in our RA patients.

## 3. Patients and Methods

During January 2007 to January 2008, about two hundred people were referred to our clinics (the rheumatology clinics of Tabriz University of Medical Sciences), only 150 patients fulfilled the criteria and finally 100 patients were enrolled in this descriptive study. We selected our patients with simple random sampling. These patients were all women and did not have a history of acute or chronic disorders such as renal failure, heart failure, or other connective tissue disorders or any established psychological disorders. We enrolled the entire patients who referred to our clinic according to the inclusion and exclusion criteria. This study was approved by the local ethics committee in November 2006; all the participants were volunteers and before the start of the study a written consent was signed by all patients. All the data were kept confidential. It was possible for every patient to quit the research study at any time during the study. RA is more prevalent in women, so we chose only women for our study. RA was diagnosed in these patients using the ACR criteria (16). Disease activity was evaluated using the DAS-28 scoring system (17). It consists of four domains (healing sense of patients according to the visual analog, number of tender, and swollen joints and CRP level). The evaluation of swollen and tender joints was done by other physicians blinded to the identity of subjects. DAS-28 is divided into four categories based on the disease activity (DAS less than 2.6 is remission, less than 3.2 is mild, 3.2-5.1 is moderate and above 5.1 is severe) (18).

GHQ-28 was used by the same authors in other quality of life studies in Iranian people (19, 20). General health questionnaire version 28 (GHQ -28) was used for evaluation of mental status. It was translated to Farsi. Validity and reliability of the questionnaire was approved previously (21-23). GHQ is a questionnaire which was used in the clinical situations for the detection of psychological morbidity. Goldberg and Hiller designed this questionnaire in 1979 (23). Diagnosing a psychological illness is not the aim of this questionnaire. Association between the psychological illness and health status of a subject is the main aim of GHQ-28. This questionnaire contains 28 questions which are reported on the scales 0 to 4. These scores ranged from 0-84 and according to the cutoff point of 22, patients were divided into two groups (with and without mental problems). Patients with psychological problems were referred to a psychologist for further evaluation with DSM-IV-TR questionnaire and results will be published very soon. Two groups were categorized as GHQ-28 score ≤ 22 and > 22. In this scaling system, a higher score, implies a more severe problem. This test has 4 axes: somatic symptoms, anxiety, sleep problems, social activities and depression. Each axis has seven questions. The final score was calculated based on the score given to these questions. The used questionnaire was designed according to the patients’ satisfaction and desire to participate. All of the obtained data were kept confidential. This study was approved by the Ethics Committee of Tabriz University of Medical Sciences. Data were described as Mean ± SD. We used independent t-test, Mann-Whitney U, Chi square and ANOVA tests for the data analysis. Correlations were detected using the Pearson coefficient test. In this study a P ≤ 0.05 was considered significant. SPSS software version 15 was used for the data analysis.

## 4. Results

One hundred women with RA were enrolled in this study. Mean age were 44.74 ± 13 and 47.40 ± 12 years in patients without and with psychological problems, respectively (P = 0.306). Disease duration were 4.08 ± 3.46 and 7.51 ± 5.47 years in patients without and with psychological problems, respectively (P = 0.001). Remission was observed in 43% of patients. Mild disease was seen in 11%, moderate in 37 % and severe in 9% of patients. DAS – 28, GHQ -28 scores and educational status are summarized in Table 1. 

Somatic symptoms were more frequent in patients with more severe disease (P = 0.002). According to Table 2 somatic symptoms were more common in patients with active form of disease (9.33 ± 1.41). Also maximum score of GHQ-28 was seen in severe disease. There was a remarkable correlation between somatic symptoms, anxiety, sleep disorders and depression with the GHQ-28 score (Table 3). 

There was a significant correlation between GHQ and disease activity (Table 4). Somatic symptoms had moderate correlation with GHQ results and the correlation was mild in the case of other parameters. According to the GHQ-28 questionnaire, 49% of patients with RA had psychological problems.

**Table 1.** Characteristics of Patients

	Without Psychological Problem	With Psychological Problem	P value
**DAS28**	2.71± 1.6	3.74 ± 1.33	0.001
**Disease activity ** ^**a**^			0.001
Remission	31	12	
Mild	5	6	
Moderate	13	24	
Severe	2	7	
**GHQ 28**	15.78 ± 3.40	31.26 ± 7.41	0.001
Somatic symptoms	5.00 ± 2.25	8.59 ± 2.36	
Anxiety and insomnia	3.47 ± 2.11	8.00 ± 3.08	
Social activities	5.33 ± 1.96	8.25 ± 2.81	
Depression	1.98 ± 1.81	6.242 ± 3.59	
**Education**			0.001
Illiterate	14	24	
Elementary school	18	18	
High school and university	19	7	

^a^ Das-28 is divided into four stages (< 2.6: remission, < 3.2: mild, 3.2-5.1: moderate, > 5.1: severe)

**Table 2.** GHQ levels And Disease Activity of RA

Disease activity	GHQ parameters				
	GHQ 28	Depression	Social Activity	Anxiety and Insomnia	Somatic Symptoms
**Remission**	19.48 ± 9.76	3.41 ± 3.16	6.12 ± 2.68	4.88 ± 3.56	5.67 ± 2.6
**Mild**	24.09 ± 9.49	4.64 ± 4.18	7.18 ± 3.68	5.64 ± 3.11	6.64 ± 3.67
**Moderate**	26.08 ± 8.79	5.22 ± 3.7	7.27 ± 2.62	6.16 ± 2.94	7.43 ± 2.59
**Severee**	28.22 ± 7.9	4 ± 2.24	7.22 ± 2.99	7.67 ± 4.8	9.33 ± 1.41
**Total DAS**	23.37 ± 9.64	4.16 ± 3.60	6.76 ± 2.81	5.69 ± 3.47	6.76 ± 2.85
**P value**	0.010	0.080	0.269	0.114	0.001

**Table 3.** Correlation Between the GHQ Score and Investigated Parameters

GHQ score	GHQ Parameters			
	Depression	Social Activity	Anxiety and Insomnia	Somatic Symptoms
**R**	0.773	0.7020	0.851	0.678
**P value**	0.001	0.001	0.001	0.001

**Table 4.** Correlations Between GHQ Parameters and the Disease Activity ^a^

Disease Activity	GHQ Parameters				
	GHQ28	Depression	Social Activity	Anxiety and Insomnia	Somatic Symptoms
**DAS**	-	-	-	-	-
R	0.329	0.188	0.196	0.237	0.394
P.value	0.001	0.610	0.510	018.0	0.001

^a^ Spearman correlation was used (P: P value and R: correlation coefficient)

## 5. Discussion

According to the GHQ -28 questionnaire results, psychological problems were seen in 49% of studied patients. The results of other available studies are different. Creed et al. showed that psychological problems are seen in 20-25 % of patients (13). Cadena showed that symptoms of depression, helplessness, disability, pain, anxiety, lower quality of life, and self-efficacy were associated with RA activity regardless of the age, sex, and duration of the disease. Symptoms of depression were directly correlated with anxiety, helplessness, pain, and disability and inversely correlated with quality of life and self-efficacy. These results indicate that RA activity significantly affects the mental health status and quality of life in this population. Accordingly, a holistic therapy regimen should guide the treatment of patients with RA (6). Michaud showed that a poor baseline health status was associated with greater mortality risk. After adjusting for the age, sex, and baseline physical component score (PCS) and mental component score (MCS), decline in PCS and health associated questionnaire (HAQ) were associated with higher risk of death. HAQ improvement was associated with reduced mortality risk from 6 months to 3 years; a similar relationship was not observed for PCS or MCS improvement. After controlling for the baseline values, changes in PCS or HAQ did not improve prediction accuracy (8). Dickens et al. studied 74 patients with RA in U.K. and showed that psychological problems were seen in 19 patients (25.7%). Among them, depression was seen in 12 patients (16.2%) and anxiety in 7 patients (9.5%) (22). Two other mental health status studies in England were performed by Fifield and Pincus and concluded that the prevalence of psychological problems in RA patients was 3-80% (23). Murphy et al. studied 80 RA patients and 21% of them had depression or anxiety (24). Zyrianova in Russia studied 68 RA patients and 37.5% suffered from depression and 44.4% had anxiety disorders (25). Sometimes there is not a significant relationship present between the GHQ-28 score and the studied axes (somatic symptoms, anxiety, sleep disorders and depression). We tried to identify the most important axis which is responsible for the higher score. The patient may have very high scores in three studied axes, but a lower score in one of them. There was a significant correlation between the GHQ score and the disease activity. Somatic symptoms had moderate correlation and in other parameters correlation was mild. El- Miedany reported in 80 RA patients that depression was seen in 62.2% and anxiety was seen in 70% of studied patients (26).

Totally, as mentioned above, the frequency of psychological problems in RA patients was between 3 and 80%. Our study showed that a remarkable percentage (almost 50%) of RA patients had a kind of psychological problem and needed further evaluation. This discrepancy may be a due to difference in sample size, cultural and geographical differences (24), and the applied screening and diagnostic methods. There are many risk factors which may affect mental status of RA patients and may affect the results. There is a significant correlation between the disease activity (according to the DAS-28) and the GHQ -28 score in RA patients. It seems that high disease activity may lead to increased frequency of psychological problems. Dickens et al showed that there is a correlation between the disease activity and the occurrence of psychological problems in RA (22). Atapoor and colleagues from the Tehran University showed that there is a close relations between the education level and occurrence of psychological problems and these problems are more usual in illiterate patients (27).

We also achieved similar results (P = 0.001). Alishiri et al. studied 411 patients with RA and showed that, disease duration, presence of underlying problems and disease activity had significant correlation with psychological problems (28). According to the above mentioned results, a correlation between disease activity and psychological problems has been reported in various studies. Optimum disease control may be effective in decreasing psychological problems in RA patients. Some patients with psychological problems did not like to undergo psychological assessment and receive treatment for that. Social stigmata associated with psychological problems is one of the most important reasons (19). Supporting patients’ family in preventing and treating mental problems is very important (27). When the disease is mildly active, diagnosing of psychological problems is difficult because some symptoms and signs are related to RA disease but not the psychological problems.

Noorbala et al. studied the mental health status of the adult population in Iran. Depression and anxiety symptoms were more prevalent than somatization and social dysfunction. Interviewing families by general practitioners revealed that rates of learning disability, epilepsy and psychosis were 1.4%, 1.2% and 0.6%, respectively. These results are comparable with ours (29). It is preferred that similar studies will be performed in the future with greater sample size. Using other questionnaires like SCL-90 and comparing the results may be important for obtaining the best results in our setting. Also it must be emphasized that all of the patients received corticosteroids during the therapy and some of these psychological findings may be related to this fact. It is better that this type of studies be performed with larger sample sizes and also such a study must be performed using various questionnaire types and results must be compared with each other. This study clearly shows that a majority of patients with RA simultaneously suffer from psychological problems. These problems are more probable when the disease is more severe or prolonged. Referral to a psychologist is recommended for these patients.
